# Detection and Genomic Characteristics of NDM-19- and QnrS11-Producing O101:H5 *Escherichia coli* Strain Phylogroup A: ST167 from a Poultry Farm in Egypt

**DOI:** 10.3390/microorganisms13081769

**Published:** 2025-07-29

**Authors:** Ahmed M. Soliman, Hazem Ramadan, Toshi Shimamoto, Tetsuya Komatsu, Fumito Maruyama, Tadashi Shimamoto

**Affiliations:** 1 Department of Microbiology and Immunology, Faculty of Pharmacy, Kafrelsheikh University, Kafr El-Sheikh 33516, Egypt; ahmed_soliman@pharm.kfs.edu.eg; 2 Hygiene and Zoonoses Department, Faculty of Veterinary Medicine, Mansoura University, Mansoura 35516, Egypt; hazem_hassan@mans.edu.eg; 3 Laboratory of Food Microbiology and Hygiene, Graduate School of Integrated Sciences for Life, Hiroshima University, Higashihiroshima 739-8528, Japan; 4 Livestock Industry Division, Bureau of Agriculture and Fisheries, Aichi Prefectural Government, Nagoya 460-8501, Japan; tetsuya_1_komatsu@pref.aichi.lg.jp; 5 Section of Microbial Genomics and Ecology, Planetary Health and Innovation Science Center (PHIS), The IDEC Institute, Hiroshima University, Higashihiroshima 739-8530, Japan; fumito@hiroshima-u.ac.jp

**Keywords:** pAMS-X3-NDM-19, *bla*_NDM-19_, IncX3, *qnrS11*, *Escherichia coli*, poultry, Egypt

## Abstract

This study describes the first complete genomic sequence of an NDM-19 and QnrS11-producing multidrug-resistant (MDR) *Escherichia coli* isolate collected from a fecal swab from a poultry farm in 2019 in Egypt. The *bla*_NDM-19_ was identified by PCR screening and DNA sequencing. The isolate was then subjected to antimicrobial susceptibility testing, conjugation and transformation experiments, and complete genome sequencing. The chromosome of strain M2-13-1 measures 4,738,278 bp and encodes 4557 predicted genes, with an average G + C content of 50.8%. M2-13-1 is classified under ST167, serotype O101:H5, phylogroup A, and shows an MDR phenotype, having minimum inhibitory concentrations (MICs) of 64 mg/L for both meropenem and doripenem. The genes *bla*_NDM-19_ and *qnrS11* are present on 49,816 bp IncX3 and 113,285 bp IncFII: IncFIB plasmids, respectively. M2-13-1 harbors genes that impart resistance to sulfonamides (*sul1*), trimethoprim (*dfrA14*), β-lactams (*bla*_TEM-1B_), aminoglycosides (*aph(6)-Id*, *aph(3′)-Ia*, *aph(3″)-Ib*, *aac(3)-IV*, and *aph(4)-Ia*), tetracycline (*tet*(A)), and chloramphenicol (*floR*). It was susceptible to aztreonam, colistin, fosfomycin, and tigecycline. The genetic context surrounding *bla*_NDM-19_ includes IS*Aba125*-IS*5*-*bla*_NDM-19_-*ble*_MBL_-*trp*F-*hp*1-*hp*2-IS*26*. Hierarchical clustering of the core genome MLST (HierCC) indicated M2-13-1 clusters with global ST167 *E. coli* lineages, showing HC levels of 100 (HC100) core genome allelic differences. Plasmids of the IncX3 group and the insertion sequence (IS*Aba125*) are critical vehicles for the dissemination of *bla*_NDM_ and its related variants. To our knowledge, this is the first genomic report of a *bla*_NDM-19_/IncX3-carrying *E. coli* isolate of animal origin globally.

## 1. Introduction

Carbapenem antibiotics (e.g., imipenem, meropenem, doripenem, and ertapenem) are considered as last-resort treatments for infections caused by multidrug-resistant bacteria [1,2]. This class exhibits a broad spectrum of activity against bacteria and improved stability against β-lactamases [1,2]. In 2021, the WHO developed the Access, Watch, Reserve (AWaRe) classification of antibiotics to help reduce bacterial resistance and improve antimicrobial stewardship (https://www.who.int/publications/i/item/2021-aware-classification, accessed on 15 January 2024). It was noted that most of the carbapenems were on the WHO watch list, while imipenem/cilastatin/relebactam and meropenem/vaborbactam were on the WHO reserve list.

The spread and emergence of resistance to carbapenems is a serious public health concern. In Enterobacteriaceae, the resistance to carbapenem antibiotics is facilitated by the production of a great number of carbapenemases, which include (i) Ambler class A β-lactamases (KPC, FRI, GES, SME), (ii) Ambler class B metallo-β-lactamases (VIM, NDM, IMP, GIM, SPM), and (iii) Ambler class D oxacillinases (OXA-type) prevalent mainly in *Acinetobacter baumannii* and *Pseudomonas aeruginosa* [1,2,3]. We have previously reported, from Egypt, (i) the first NDM-5-producing *E. coli* clinical isolate [4], (ii) the coexistence of NDM-5 and NDM-4 in *Klebsiella pneumoniae* from a 6-month-old infant [5], (iii) the first NDM-1-producing-*Providencia stuartii* isolates in an African burn unit [3], (iv) two NDM-5 and OXA-181 co-producing *E. coli* clinical isolates [3], and (v) the first whole genome sequence of *E. coli* clinical isolate co-carrying *bla*_NDM-1_ and *bla*_OXA-244_ [6]. Additionally, we indicated the elevated incidence of carbapenemase-producing Gram-negative bacteria in Egypt, with OXA-48-like and NDM-1 being the most prevalent [7].

As of 19 January 2023, 48 variants of *bla*_NDM_ are accessible in the Bacterial Antimicrobial Resistance Reference Gene Database (https://www.ncbi.nlm.nih.gov/pathogens/refgene/#NDM, accessed on 19 January 2023). The New Delhi metallo-β-lactamase (NDM) variant, NDM-19, differs from NDM-1 and NDM-7 by three (Asp130Asn, Met154Leu, and Ala233Val) and a single amino acid substitution (Ala233Val), respectively, leading to decreased susceptibility towards carbapenems and extended-spectrum cephalosporins [2,8,9,10]. NDM-19 was previously identified from human clinical *E. coli* and/or *K. pneumoniae* isolates from different countries, including China, Egypt, Lebanon, and Switzerland [2,8,9,10].

El-Kholy et al., 2021 reported a high prevalence of carbapenem-resistant Enterobacteriaceae (CRE) (13.8–100% of *E. coli* and 35–100% of *K. pneumoniae*) in Egypt [11]. In the United Arab Emirates, an analysis was conducted on a total of 381,535 non-repetitive Enterobacteriaceae from 2010 to 2021, with CRE accounting for 3.8%, representing 74 different species [12]. Meanwhile, 5% of CRE was identified in a recent surveillance study from Saudi Arabia [12]. In Lebanon, the CRE rate was 2.8% in 2022, while in Jordan, it was 1.6% in 2015 [12].

CRE is a group of Gram-negative Enterobacteriaceae that are resistant to carbapenems and can cause serious illnesses such as urinary tract infections, sepsis, and pneumonia [13]. CRE can be found in various settings, including hospitals, the environment, and animals, highlighting a zoonotic transmission [13]. Chickens are raised close to humans, such as in backyards and small-scale farms, and can act as a critical reservoir of CRE. An increased incidence of CRE has been observed in poultry farms, predominantly in *E. coli, K. pneumoniae*, and *P. mirabilis*, carrying *bla*_NDM_ genes, with *bla*_NDM-1_ and *bla*_NDM-5_ being the most prevalent [14].

The genomic features of NDM-19-producing *E. coli* in Egypt remain scarce. Therefore, in the current study, we aimed to illustrate the first complete genomic sequence, produced by the Oxford Nanopore and Illumina MiniSeq sequencing techniques, of the NDM-19-producing *E. coli* strain from a poultry farm in Egypt.

## 2. Materials and Methods

### 2.1. Isolation and Identification of E. coli M2-13-1

The *E. coli* strain M2-13-1 was isolated from a fecal sample collected from a poultry farm in March 2019 in Sidi Ghazy City, Kafr El-Sheikh province, Egypt. The farm has healthy commercial chickens aged 15 to 30 days. The fecal sample was first incubated in Luria-Bertani (LB) (Lennox) broth at 37 °C for 18–24 h. After enrichment, the culture was plated onto MacConkey agar with 2 µg/mL meropenem. The resulting isolate showed resistance to carbapenems. The 16S ribosomal RNA gene amplification and sequencing using primers 27F and 1492R was conducted to identify the isolate [3]. PCR screening was performed for several carbapenemase encoding genes (NDM, VIM, IMP, KPC, OXA-48-like), as previously reported [3]. NDM variants were detected by PCR screening and DNA sequencing by NDM-F (5′-GGTTTGGCGATCTGGTTTTC-3′) and NDM-R (5′-CGGAATGGCTCATCACGATC-3′), NDM-WHF (5′-ATGGAATTGCCCAATATTATGCACC-3′), and NDM-WHR (5′-TCAGCGCAGCTTGTCGG-3′), amplifying PCR products of 621 and 813 base pairs (bp), respectively [3,15]. Sanger sequencing confirmed the presence of the NDM-19 variant. Consequently, the strain was submitted for whole-genome sequencing.

### 2.2. Minimum Inhibitory Concentration (MIC) of Various Antimicrobial Agents Against M2-13-1

MIC of various antimicrobial agents against M2-13-1 was determined according to the standards and interpretive criteria defined by the Clinical and Laboratory Standards Institute (CLSI, document M100-S24). The following antibiotics were used: aztreonam, cefotaxime, chloramphenicol, ciprofloxacin, colistin, ertapenem, fosfomycin, kanamycin, meropenem, tetracycline, and tigecycline. *E. coli* ATCC 25922 was used as a control.

### 2.3. Conjugation and Transformation Experiments

To assess the mobility of the plasmid carrying *bla*_NDM-19_, a filter-mating conjugation was achieved using M2-13-1 as the donor and the azide-resistant *E. coli* strain J53 as a recipient [3]. An *E. coli* strain carrying NDM-5 on an IncX3 plasmid from our collection was used as a control. The transconjugants were selected on LB agar plates containing 50 μg/mL ampicillin and 100 μg/mL sodium azide or 1 μg/mL imipenem and 100 μg/mL sodium azide [3]. For transformation, plasmid DNA was extracted by a standardized alkaline lysis method, as previously described [3]. Plasmids were CaCl_2_-transformed into *E. coli* DH5α. Transformants were selected on LB agar plates plus 1 μg/mL meropenem or 100 μg/mL ampicillin. Colony-direct PCR was accomplished utilizing the primers NDM-F and NDM-R to verify the transmission of the *bla*_NDM-19_-containing plasmid [15].

### 2.4. Complete Genome Sequencing (WGS) and Analysis

The total genomic DNA (gDNA) of the *E. coli* M2-13-1 strain was prepared from an overnight bacterial culture using a standard proteinase K method, as previously reported [16]. DeNovix (Wilmington, DE, USA), Bioanalyzer (Agilent Technologies, Santa Clara, CA, USA), and a Microdrop (Thermo Scientific™, Multiskan™ Sky Microplate Spectrophotometer, Waltham, MA, USA) were utilized to assess the quality of the isolated gDNA. For Illumina MiniSeq sequencing, a Nextera DNA Flex Library Prep Kit (Illumina, San Diego, CA, USA) was employed to construct the library. For Oxford Nanopore sequencing, the Rapid Barcoding Sequencing kit (SQK-RBK004) (Oxford Nanopore Technologies, Oxford, UK) was used to construct the DNA library, which was then loaded onto a flow cell (FLO-MIN106) and sequenced for 48 h with GridION (Oxford Nanopore Technologies). Flye v2.6 (https://github.com/fenderglass/Flye) was used to perform the hybrid assembly of both Illumina short reads and Nanopore long reads. The genome assembly was further refined using Pilon-v1.23 [17]. Annotation was performed using DFAST (https://dfast.nig.ac.jp/) [18]. The complete genome of the *E. coli* M2-13-1 strain was examined using ResFinder-3.2, PlasmidFinder-2.0, SerotypeFinder-2.0, MLST 2.0, pMLST-2.0, and CHTyper-1.0, accessible at the Center for Genomic Epidemiology (http://www.genomicepidemiology.org/). Phylogenetic grouping was identified using the Clermont Typing scheme available at (http://clermontyping.iame-research.center/). A circular comparison between the complete sequence of pAMS-X3-NDM-19 and the highly similar plasmids identified via a NCBI nucleotide BLAST (BLASTn) (https://blast.ncbi.nlm.nih.gov/Blast.cgi) search was conducted using the BRIG tool [19]. The genetic environment of *bla*_NDM-19_ was illustrated through a BLASTn search and ISFinder (https://www-is.biotoul.fr/blast.php) to detect insertion sequence (IS) elements. Visualization and comparison of the genetic environment of *bla*_NDM-19_ in pAMS-X3-NDM-19 was accomplished with the EasyFig_win_2.1 tool (http://mjsull.github.io/Easyfig/). The virulome of the NDM-19-producing *E. coli* strain M2-13-1 was predicted using the virulence factor database, a JavaScript-rich interface with VFanalyzer (http://www.mgc.ac.cn/VFs/).

For a WGS-based phylogenetic analysis, fastq files of the sequenced *E. coli* isolate were imported into Enterobase (https://enterobase.warwick.ac.uk/, version 1.2.0) and compared to publicly available genomes of ST167 *E. coli* (*n* = 198) that were randomly selected to represent different sources and countries, using single-nucleotide polymorphisms (SNPs) and hierarchical clustering (HierCC) of core genome (cg) MLST. Different sets of hierarchical clusters (HCs) were defined in Enterobase to cluster bacterial genomes based on variations in core genomic loci across bacteria [20]. The present study isolates and selected isolates from Enterobase were mapped to the *E. coli* K-12 MG1655 reference genome for SNPs analysis.

### 2.5. Nucleotide Sequence Accession Numbers

The complete genome sequence of the *E. coli* M2-13-1 strain was submitted to DDBJ/ENA/GenBank under BioProject ID: PRJNA946682.

## 3. Results and Discussion

### 3.1. Minimum Inhibitory Concentration (MIC) of Various Antimicrobials Against M2-13-1

The 16S ribosomal RNA gene sequence identified the strain as belonging to *E. coli*. Broth microdilution exhibited that M2-13-1 was resistant to ceftriaxone, chloramphenicol, meropenem, ertapenem, kanamycin, ciprofloxacin, and tetracycline. The strain showed sensitivity to aztreonam, colistin, tigecycline, and fosfomycin (Table 1). PCR screening and DNA sequencing recognized the existence of the metallo-β-lactamase genes, *bla*_NDM-19_.

### 3.2. Genomic Characterization of E. coli M2-13-1

The complete genome of *E. coli* M2-13-1 was obtained from high-quality hybrid assemblies after the combination of Illumina short reads and Nanopore long reads. The assembly was adequate for circularizing the chromosome and the plasmids. The final polished assembly of M2-13-1 comprised 10 contigs totaling 5.13 Mb with an N50 of 4.74 Mb (L50 = 1). The mean sequencing depth was ~85×, and the overall GC content was 50.7%. A total of 5010 coding sequences, 22 rRNAs, 90 tRNAs, and 2 CRISPRs were predicted. The chromosome of the *E. coli* M2-13-1 strain was 4,738,278 bp in size with an average GC content of 50.8% (Table 2). The MLST2.0 server revealed that M2-13-1 belonged to ST167 [allelic profile (*adk* 10, *fumC* 11, *icd* 8, *purA* 13, *gyrB* 4, *recA* 2, and *mdh* 8)]. SerotypeFinder-2 and ClermonTyping servers revealed that M2-13-1 belonged to serotype O101:H5 and phylogroup A, respectively.

ST167 *E. coli* producing different NDM enzymes were reported from different countries, including China (NDM-1 and NDM-7), Egypt (NDM-1 and NDM-5), Guatemala (NDM-1), Italy (NDM-5), and Thailand (NDM-5) [21,22,23].

Although the strain was isolated from a poultry farm fecal sample, it still lacks the virulence genes necessary for classifying it as an avian pathogenic *E. coli* (APEC) genetic pathotype. According to Johnson et al., 2008, an isolate is often considered APEC if it carries five or more of the following virulence genes: *iroN*, *ompT*, *hlyF*, *iss*, *iutA* [24]. M2-13-1 is not APEC because it only carries *iss* and lacks the other four genes. Based on the resistance gene profile (NDM-19 on IncX3 plasmid) and ST167, this strain is likely associated with human infection and may belong to the ExPEC pathotype, as previously reported, suggesting transmission between animals and humans and vice versa [25].

### 3.3. Plasmidome and Resistome of E. coli M2-13-1

The strain carried four different plasmids belonging to various Inc groups, including IncFII: IncFIB, IncI1-Iγ, IncY, and IncX3, ranging in size from 49,816 bp to 113,285 bp (Table 2). Additionally, the strain carried 13 different acquired antimicrobial resistance genes potentially conferring resistance to quinolone (*qnrS11*), tetracycline [*tet*(A)], trimethoprim (*dfrA14*), β-lactams (*bla*_NDM-19_ and *bla*_TEM-1B_), aminoglycosides [*aac(3)-IV*, *aph(3″)-Ib*, *aph(4)-Ia*, *aph(6)-Id*, and *aph(3′)-Ia*], macrolides [*mdf(A)*], chloramphenicol and florfenicol (*floR*), and sulfonamides (*sul2*) (Table 2). The quinolone resistance-determining region was found to be mutated (*parC*: S80I, *parE*: S458A, *gyr*A: S83L, and D87N) (Table 2), conferring resistance to ciprofloxacin and nalidixic acid.

### 3.4. Identification of a bla_NDM-19_/IncX3 Plasmid in an Egyptian E. coli Poultry Strain

New Delhi metallo-β-lactamase (NDM) is a widely distributed Ambler class B carbapenemase that confers high-level resistance to all β-lactams, except aztreonam, and is found in various Gram-negative bacteria from different sources, including humans, animals, and the environment [26]. NDM-19 was first detected in an *E. coli* strain isolated from an Egyptian patient in a Swiss hospital [8]. Subsequently, NDM-19 was identified on an IncX3 plasmid in a carbapenem-resistant (CR) ST15 *K. pneumoniae* strain isolated from a sputum swab of a patient with chronic obstructive pulmonary disease in China [9]. Additionally, an Egyptian clinical CR *K. pneumoniae* ST353 was reported to carry *bla*_NDM-19_ in 2018 [2]. Moreover, nine NDM-19-positive *E. coli* clinical strains were reported in Northern Lebanon, isolated between 2015 and 2019 [10].

**Figure 1:**
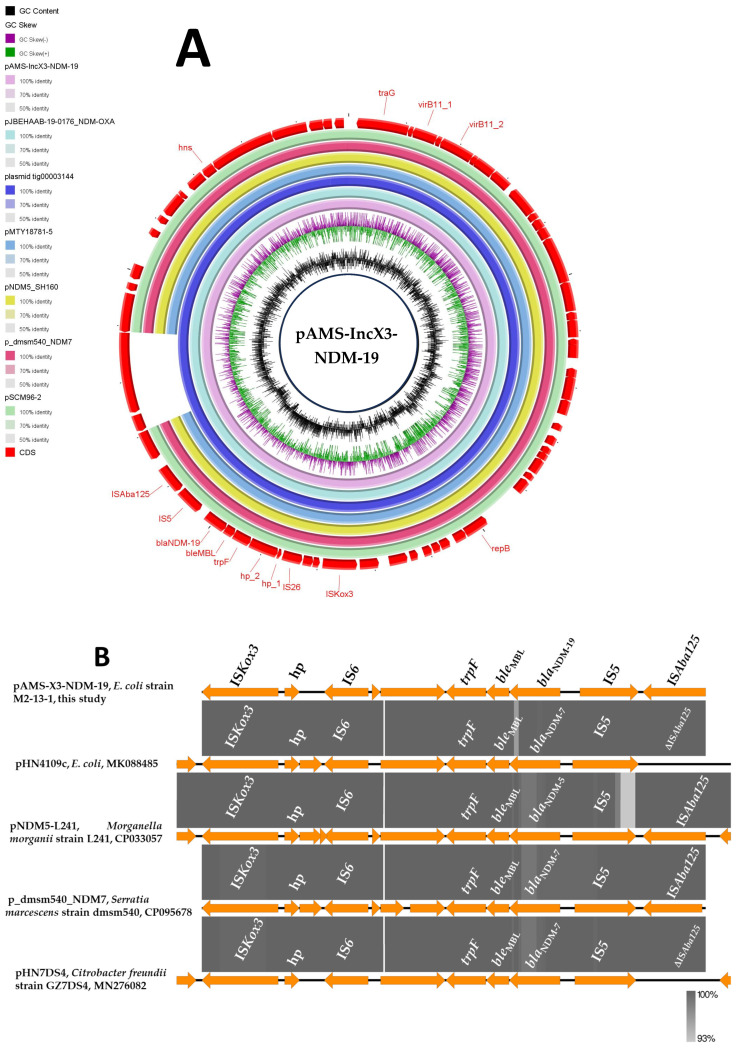
Schematic representation of IncX3 plasmid carrying *bla*_NDM-19_ (**A**), and the genetic environment of *bla*_NDM-19_ (**B**) identified from the genomic sequence of *E. coli* strain M2-13-1 analyzed in this study. In panel A, six IncX3 plasmids, pJBEHAAB-19-0176_NDM-OXA, plasmid tig00003144, pMTY18781-5, pNDM5_SH160, p_dmsm540_NDM7, and pSCM96-2 carrying *bla*_NDM_ (accession no. AP028870.1, CP021682.1, AP023210.1, CP053295.1, CP095678.1, and CP028718.1, respectively) have been detected from NCBI GenBank and were included in the figure. The whole sequence of pAMS-IncX3-NDM-19 was used as the reference. The external ring represents the annotation of pAMS-IncX3-NDM-19. The plasmids were included in the following order: pAMS-IncX3-NDM-19 (identified in this study), pJBEHAAB-19-0176_NDM-OXA, plasmid tig00003144, pMTY18781-5, pNDM5_SH160, pSCM96-2, p_dmsm540_NDM7, and pAMS-X3-NDM-19.

*bla*_NDM-19_ was located on the plasmid pAMS-X3-NDM-19. pAMS-X3-NDM-19 is an IncX3 plasmid of 49,816 bp in size encoding 64 CDS with an average G + C content of 47.3% (Figure 1 and Table 2). Plasmids of IncX3 replicons (i) have narrow host range [27]; (ii) have been frequently identified in *Enterobacteriaceae* of environmental, clinical, and animal origin [27]; (iii) are featured by carrying carbapenemase-encoding genes (e.g., *bla*_NDM_, *bla*_KPC_, and *bla*_OXA-181_) [27]; and (iv) are considered as a critical vehicle for the transmission of *bla*_NDM_ and its rare new variants with the significant role of the insertion sequence (IS*Aba125*) [27].

A BLASTn search with the entire pAMS-X3-NDM-19 sequence query identified that it has high similarity to other IncX3 plasmids carrying *bla*_NDM_. For instance, pAMS-3-NDM-19 showed more than 99.9% sequence identity to *E. coli* plasmids (i) plasmid tig00003144 carrying *bla*_NDM-7_ (100% coverage; GenBank: CP021682.1), (ii) *bla*_NDM-5_-carrying pMTY18781-5 isolated from human stool in Japan in 2018 (92% coverage; GenBank: AP023210.1), and (iii) *bla*_NDM-5_- and *bla*_OXA-181_-carrying pJBEHAAB-19-0176_NDM-OXA isolated from human stool in Japan in 2020 (100% coverage; GenBank: AP028870.1) (Figure 1A). In addition, pAMS-X3-NDM-19 has >99.9% identity to *bla*_NDM-19_-bearing *K. pneumoniae* IncX3 plasmid pSCM96-2 isolated from a sputum sample in China in 2017 (92% coverage; GenBank: CP028718.1), *bla*_NDM-7_-bearing *Serratia marcescens* IncX3 plasmid p_dmsm540_NDM7 isolated from a blood sample in Bangladesh in 2017 (92% coverage; GenBank: CP095678.1), and *Salmonella enterica* subsp. *enterica* serovar Typhimurium plasmid pNDM5_SH160 carrying *bla*_NDM-5_ (92% coverage; GenBank: CP053295.1) (Figure 1A).

The results of conjugation experiments showed that the pAMS-X3-NDM-19 plasmid was not successfully transferred to *E. coli* J53 after multiple attempts, despite the successful transfer of the control NDM-5 carrying IncX3 plasmid. Most IncX3 plasmids are capable of conjugation, with a frequency ranging from 10^−9^ to 10^−2^ [21]. Several reports described the impaired conjugation ability of IncX3 plasmids (reviewed in [21]). In some cases, this is caused by the destruction or deletion of conjugation genes. However, other IncX3 plasmids remain non-conjugative despite having the entire conjugation backbone, indicating additional regulatory mechanisms. For example, plasmid pWLK-NDM (GenBank: CP038280) was isolated from urban river debris in China and is non-conjugable due to disruption of the *pilX3*-*4* gene, even though the conjugation genes are present [27]. Furthermore, a recent study identified two key factors controlling IncX3 plasmid conjugation: H-NS, a putative DNA-binding protein, inhibits conjugation by directly repressing the expression of the activator PrfaH, whose deletion eliminates conjugative transfer [28].

The genetic environment of *bla*_NDM-19_ was as follows: IS*Aba125* (IS*30* family)-IS*5* (IS*5* family)-*bla*_NDM-19_-*ble*_MBL_ (bleomycin resistance gene)*-trpF* (phosphoribosylanthranilate isomerase)-*hp*-*hp*-IS*26* (IS*6* family)*-hp*-IS*Kox3* (IS*L3* family). A BLASTn search using the immediate genetic vicinity of *bla*_NDM-19_ identified the similarity to other sequences (100% query coverage and ≥99.94% sequence identity) from different organisms, including *E. coli* plasmid pHN4109c (GenBank: MK088485.1), plasmid pKpN01-NDM7 (GenBank: CP012990.1) in clinical *K. pneumoniae* strain KpN01 from Canada, plasmid p_dmsm540_NDM7 (GenBank: CP095678.1) in clinical *S. marcescens* strain dmsm540 from Bangladesh, plasmid pHN7DS4 (GenBank: MN276082.1) from *Citrobacter freundii* strain GZ7DS4 isolated from a dog in China, and plasmid pNDM5-L241 (GenBank: CP033057.1) from clinical *Morganella morganii* strain L241 detected in China (Figure 1B).

### 3.5. Detection of Other Multidrug-Resistant (MDR) Plasmids from E. coli M2-13-1

**Figure 2:**
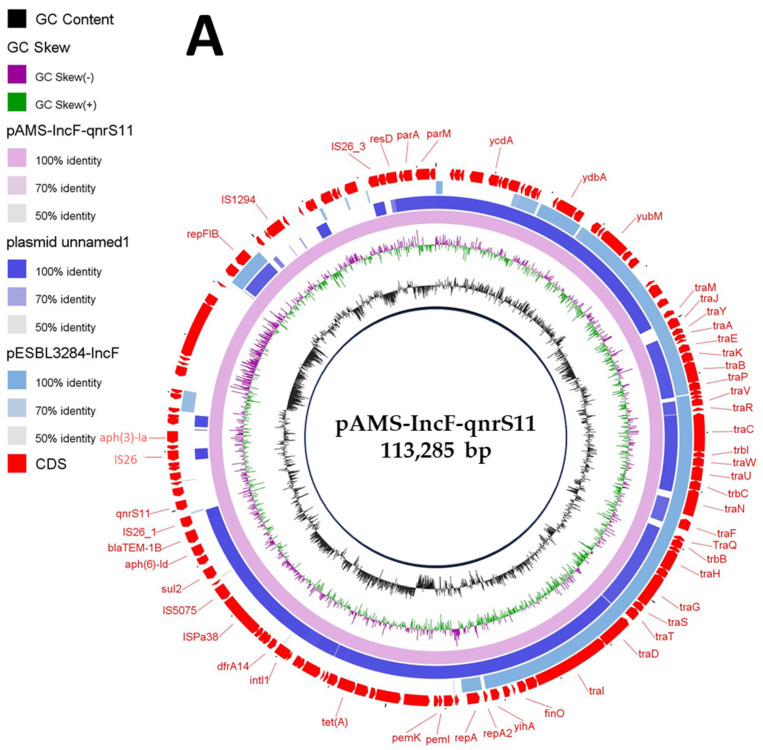

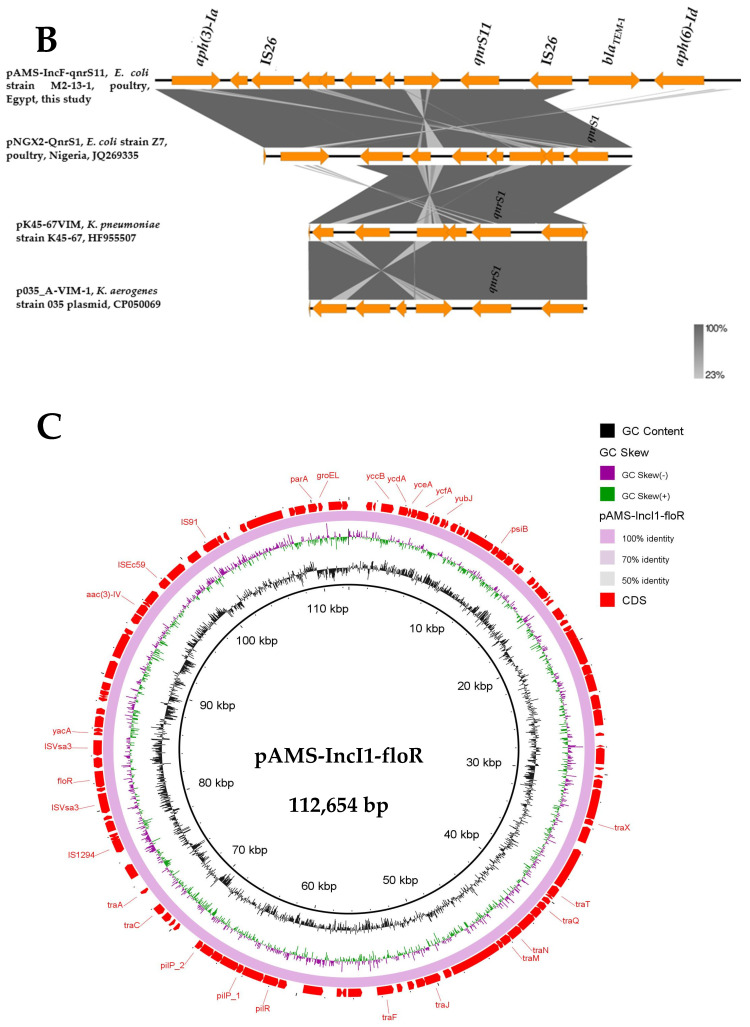
Schematic representation of IncF plasmid carrying *qnrS* (**A**), the genetic environment of *qnrS11* (**B**), and IncF plasmid carrying *floR* (**C**) identified from the genome sequences of *E. coli* strain M2-13-1 analyzed in this study. In panel A, two IncF plasmids, plasmid unnamed1 (GenBank: CP020510.1), and plasmid pESBL3284-IncF (GenBank: MW390540.1) have been detected from NCBI GenBank and were included in the figure. The whole sequence of pAMS-IncF-*qnrS11* was used as the reference. The external ring represents the annotation of pAMS-IncF-*qnrS11*. The plasmids were included in the following order: pAMS-IncF-*qnrS11* (identified in this study), plasmid unnamed1, and plasmid pESBL3284-IncF.

Here, MDR plasmids is defined as plasmids carrying genes conferring resistance to multiple antibiotics and can be transferred by horizontal gene transfer (HGT). *E. coli* M2-13-1, detected in the current study, contained another two MDR plasmids [pAMS-IncF-qnrs11 (∼113 Kb IncFII: IncFIB) (Figure 2A,B) and pAMS-IncI1-floR (∼113 Kb IncI1-Iγ) (Figure 2C)] carrying eight (*aph(6)-Id*, *aph(3′)-Ia*, *aph(3″)-Ib*, *qnrS11*, *dfrA14*, *sul2*, *tet*(A), *bla*_TEM-1B_) and four (*aac(3)-IV*, *aph(4)-Ia*, *sul2*, *floR*) resistant genes, respectively (Table 2, Figure 2).

IncF plasmids are (i) one of the most commonly detected plasmid types in *Enterobacteriaceae*, (ii) frequently of low copy number, (iii) MDR with more than 100 kb in size, and (iv) multireplicon to enhance the replication initiation (FII, FIA, and FIB) [29]. A BLASTn search with the whole pAMS-IncF-qnrs11 sequence query identified that it has more than 99.1% identity to plasmid unnamed1 isolated from *E. coli* strain 165 isolated from a patient in the USA in 2015 (76% coverage; GenBank: CP020510.1), and 98.7% identity to plasmid pESBL3284-IncF *E. coli* strain ESBL3284 (46.8% coverage; GenBank: MW390540.1) (Figure 2A). pAMS-IncF-qnrs11 was successfully transferred by conjugation to *E. coli* J53. The transconjugant showed resistance to kanamycin (MIC, >256 μg/mL) and tetracycline (MIC, 64 μg/mL) but showed sensitivity to aztreonam (MIC, <2 μg/mL), ciprofloxacin (MIC, 0.5 μg/mL), chloramphenicol (MIC, 4 μg/mL), ceftriaxone (MIC, <2 μg/mL), colistin (MIC, <0.5 μg/mL), meropenem (MIC, <0.5 μg/mL), ertapenem (MIC, <0.5 μg/mL), tigecycline (MIC, <0.5 μg/mL), and fosfomycin (MIC, 4 μg/mL).

The genetic context of *qnr*S11 was *aph(6)*-Id-*bla*_TEM-1B_-IS*26*-*qnr*S11-a gene encoding TniR protein-*hp*-*hp*-IS*26* (Figure 2B)*. qnrS11* was found between two copies of IS*26* in a transposon-like organization, which might be involved in its mobilization. A BLASTn search using the genetic environment of *qnrS11* identified the similarity to other sequences (≥99.89% sequence identity) from (i) plasmid pNGX2-QnrS1 identified from *E. coli* strain Z7 isolated from poultry in Nigeria (84% coverage; GenBank: JQ269335), (ii) plasmid pK45-67VIM identified from *K. pneumoniae* strain K45-67, (56% coverage; GenBank: HF955507), and (iii) plasmid p035_A-VIM-1 detected from *K. aerogenes* strain 035 (72% coverage; GenBank: CP050069). QnrS11 was previously reported on an IncFII plasmid in an environmental quinolone-resistant *E. coli* strain [30].

### 3.6. Analysis of the Virulome and Heavy Metal Resistance Genes

Numerous virulence-associated genes have been predicted, illustrating the pathogenicity potential associated with the consumption of contaminated chicken meat (Table 3). *E. coli* common pilus (ECP) (*ecpABCDE*), *E. coli* laminin-binding fimbriae (*elfACD*), *eaeH*, hemorrhagic *E. coli* pilus (*hcpABC*), and type I fimbriae (*fimDF*) were detected from the M2-13-1 chromosome and were mainly associated with adherence (Table 3). Type IV pili (*pilQRSVW*) was predicted from pAMS-IncI1-floR. It was suggested that pathogenic *E. coli* strains might use ECP to simulate commensal *E. coli* with an advantage for evasion of the immune system after the host colonization [31]. EhaB autotransporter protein promotes biofilm formation and binds to collagen I and laminin, inducing a serum IgA response in *E. coli* O157:H7-challenged cattle [32,33]. The invasion genes *ibeBC* contribute to *E. coli* invasion of brain microvascular endothelial cells (BMECs) or the blood–brain barrier, causing positive culture of the cerebrospinal fluid [34]. The epithelial cell adherence gene *tia* is an outer membrane protein implicated in the initial infection in ETEC strain H10407 and was detected in M2-13-1 analyzed in the current study [35]. Additionally, M2-13-1 is predicted to have genes encoding a type VI secretion system (T6SS), which penetrates epithelial cells, replicates within macrophages, and results in fatal illness in chicks [36].

### 3.7. Clonal Relationship of ST167 E. coli Isolate

**Figure 3:**
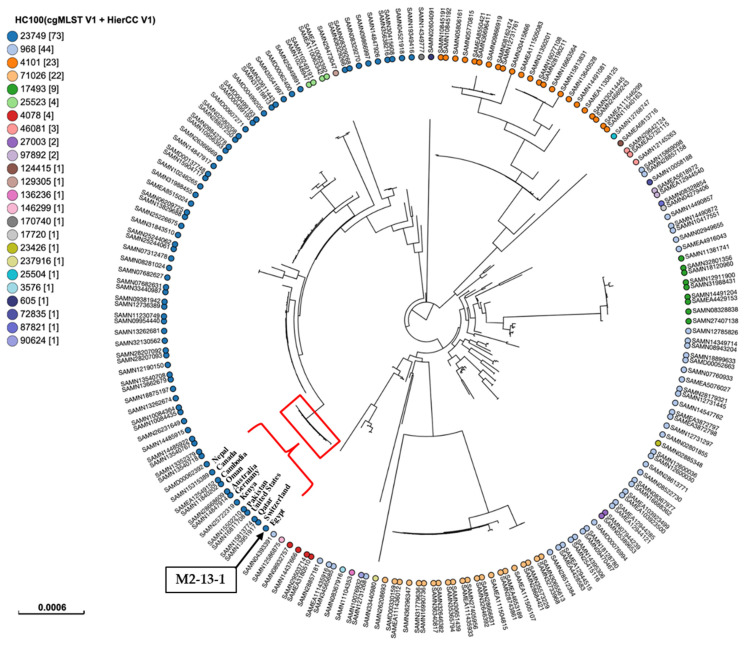
SNPs and hierarchical clustering of cgMLST (HierCC) of the present *E. coli* isolate ST167 M2-13-1 with the publicly available *E. coli* isolates ST167 sourced from different hosts in EnteroBase (https://enterobase.warwick.ac.uk/). Tip labels indicate the cgMLST pattern HC100 (allelic differences no more than 100) for ST167. The numbers in brackets indicate the number of isolates assigned to each HC100 cluster. The position of M2-13-1 was marked with a black box and a black arrow. M2-13-1 was grouped with several isolates from different countries and marked with a red triangle and curly bracket.

To determine the clonal relatedness between our isolate and global lineages of ST167, we performed a phylogenetic analysis based on SNPs and HierCC of cgMLST (Figure 3). The isolate was compared to 198 publicly available isolates of ST167 sourced from different hosts. Metadata of all strains were mentioned in Supplementary Table S1, associated with this article. The findings revealed the classification of isolates into 24 patterns at the HC100 level, differing by 100 core genomic loci. Of these HC100 patterns, four patterns: HC100|23749, HC100|968, HC100|4101, and HC100|71026 were recognized as the major patterns, assigned to 73, 44, 23, and 22 isolates, respectively. Our isolate shared the same HC100 pattern, HC100|23749, with other ST167 *E. coli* isolates from various countries, including the United States, Canada, Switzerland, Germany, the United Kingdom, Finland, Qatar, Oman, Pakistan, India, Nepal, Bangladesh, Kenya, Australia, Cambodia, China, Thailand, and Japan. The circulation of similar bacterial clones across different countries highlights the importance of implementing WGS-based phylogeny for routine epidemiological surveillance of infectious diseases, particularly those caused by clones that cross borders [37].

## 4. Conclusions

Here, we reported, to our knowledge, the first (i) detection of NDM-19-producing *E. coli* strain of chicken origin globally, and (ii) complete genome sequence of a carbapenem-resistant *E. coli* strain producing NDM-19 from Africa and the Middle East. This study demonstrated that breeding animals may serve as a crucial reservoir of multidrug-resistant bacteria carrying plasmids encoding resistance genes (*bla*_NDM-19_) to clinically important antibiotics, including imipenem and meropenem. Additionally, the finding of these *E. coli* clones from Egyptian poultry is disturbing and causes food security and public health concerns, as they might be transmitted to individuals. Plasmids of the IncX3 type and the two insertion sequences (IS*Aba125* and IS*26*) might be important tools for the spread of *bla*_NDM-19_. A survey for *bla*_NDM-19_ and other carbapenemase-encoding genes in animals is essential in Egypt to define its incidence and to stop its spread. Infection prevention and control policies, antimicrobial stewardship and surveillance plans, as well as the careful use of β-lactams in humans and animals are effectively required. The major limitation of the study is its dependence on a single bacterial strain for experimental analysis, restricting the broader application of the results.

## Figures and Tables

**Table 1:** MICs of antimicrobials for NDM-19- and QnrS11-producing *Escherichia coli* strain and its transformant/transconjugants isolated in this study.

Antibiotic	MIC (mg/L) ^a^ for Strain:			
	M2-13-1 (Wild type) ^b^	M2-13-1 TS1 (*E. coli* DH5α + pAMS-X3-NDM-19)	M2-13-1 TC (*E. coli* J53 + pAMS-IncF-qnrs11)	*E. coli* ATCC 25922
**Aztreonam**	<2	<2	<2	<2
**Ceftriaxone**	**>256**	**64**	<2	<2
**Meropenem**	**64**	**4**	<0.5	<2
**Doripenem**	**16**	**4**	ND	<2
**Ertapenem**	**256**	**8**	<0.5	<2
**Tetracycline**	**64**	<2	**64**	<2
**Ciprofloxacin**	**128**	<2	0.5	<2
**Kanamycin**	**>256**	<2	**>256**	<2
**Gentamicin**	**32**	<2	ND	<2
**Chloramphenicol**	**256**	<2	4	4
**Fosfomycin**	64	<4	4	64
**Colistin**	<0.5	<0.5	<0.5	<0.5
**Tigecycline**	1	1	<0.5	1

^a^ Resistance to the antibiotics is given in boldface. Interpretation was performed according to the CLSI guidelines (document M100-S24). TC, transconjugant; TS, transformant; ND, not determined. ^b^ Donor.

**Table 2:** Features of the chromosome and the plasmids of *E. coli* strain M2-13-1 isolated from a poultry farm in Egypt.

Sample	Size (bp)	GC%	No. of CDSs	MLST or pMLST	Incompatibility Group	Antimicrobial Resistance Genes	QRDR Point Mutations	Virulence Genes
**Chromosome**	4,738,278	50.8	4557	ST167	NA	ND	*parC*: S80I, *parE*: S458A, *gyrA*: S83L, and D87N	*gad*, *terC*, *hra*, *capU*, *iss*, *csgA*, *hha*, *hlyE*, *hra*, *nlpI*, *yehB*, *yehC*, *yehD*
**pAMS-F-qnrs11**	113,285	51.5	130	F34: A-: B53	IncFII: IncFIB	*aph(6)-Id*, *aph(3′)-Ia*, *aph(3″)-Ib*, *qnrS11*, *dfrA14*, *sul2*, *tet*(A), *bla*_TEM-1B_	NA	*anr, traJ*, *traT*
**pAMS-IncI1-floR**	112,645	51.1	119	ST3	IncI1-Iγ	*aac(3)-IV*, *aph(4)-Ia*, *sul2*, *floR*	NA	*cib*
**pAMS-IncY**	106,741	47.6	118	ND	IncY	ND	NA	ND
**pAMS-X3-NDM-19**	49,816	47.3	64	ND	IncX3	*bla*_NDM-19_	NA	ND

Abbreviations: *gad*, glutamate decarboxylase; *terC*, tellurium ion resistance proetin; *hra*, heat-resistant agglutinin; *capU*, hexosyltransferase homolog; *iss*, increased serum survival; *traT*, outer membrane protein complement resistance; *csgA*, curlin major subunit CsgA; *hha*, hemolysin expression modulator Hha (previous *rmoA*); *traJ*, protein TraJ (positive regulator of conjugal transfer operon); *cib*, colicin B; *hlyE*, avian *E. coli* haemolysin; *hra*, heat-resistant agglutinin; *nlpI*, lipoprotein NlpI precursor; *yehB*, usher (YHD fimbriael cluster); *yehC*, chaperone (YHD fimbriael cluster); *yehD*, major pilin subunit (YHD fimbriael cluster); NA, not applicable; ND, not determined.

**Table 3:** The virulome of the NDM-19-producing *E. coli* strain M2-13-1 according to the virulence factor database (http://www.mgc.ac.cn/VFs/) ^a^.

	Chromosome	pAMS-IncI1-floR
**Adherence**	*E. coli* common pilus (*ecpABCDE*) *E. coli* laminin-binding fimbriae (*elfACD*) *eaeH* Hemorrhagic *E. coli* pilus (*hcpABC*) Type I fimbriae (*fimDF*)	Type IV pili (*pilQRSVW*)
**Autotransporter**	EhaB autotransporter protein (*ehaB*)	NA
**Invasion**	Invasion of brain endothelial cells (*ibeBC*) Epithelial cell adherence (*tia*)	NA
**Non-LEE encoded TTSS effectors**	*espL1*, *espL4*, *espR1*, *espX1*, *espX4*, *esp*X5, *espY1*	NA
**Secretion systems**	ACE T6SS (*aec15*, *aec16*, *aec17*, *aec18*, *aec19*, *aec22*, *aec24*, *aec25*, *aec26*, *aec27/clpV, aec28*, *aec29*, *aec30*, *aec31*, aec*32*)	NA
**Toxins**	Hemolysin/cytolysin A (*hlyE/clyA*)	NA
**Antiphagocytosis**	capsule	NA
**Fimbrial adherence determinants**	*stjC*	NA
**Immune evasion**	Exopolysaccharide (*galE*)	NA
**Serum resistance**	LPS rfb locus (*rmlD*)	NA

^a^ NA, not applicable; LPS, lipopolysaccharide.

## Data Availability

The complete genome sequence of the *E. coli* M2-13-1 strain was submitted to DDBJ/ENA/GenBank under BioProject ID: PRJNA946682.

## Supplementary Materials

The following Supporting Information can be downloaded at: https://www.mdpi.com/article/10.3390/microorganisms13081769/s1, Supplementary Table S1: Metadata of the strains used in the phylogenetic tree.
